# Why the “Visitor Effect” Is Complicated. Unraveling Individual Animal, Visitor Number, and Climatic Influences on Behavior, Space Use and Interactions With Keepers—A Case Study on Captive Hornbills

**DOI:** 10.3389/fvets.2020.00236

**Published:** 2020-04-28

**Authors:** Paul E. Rose, Jake S. Scales, James E. Brereton

**Affiliations:** ^1^Department of Psychology, University of Exeter, Exeter, United Kingdom; ^2^WWT Slimbridge, Gloucester, United Kingdom; ^3^Sparsholt College, Winchester, United Kingdom

**Keywords:** *Ceratogymna atrata*, black-casqued hornbill, bird behavior, visitor effect, keeper effect, zoo animal welfare

## Abstract

A “visitor effect” on zoo-housed species has been documented since the 1970s, with research focused on mammals (specifically primates). To broaden our understanding of the “visitor effect” in a non-mammal, we conducted a case study on a pair of hornbills, recording behavior and aviary use alongside of visitor and keeper presence. Temperature and humidity were significant predictors of visitor number, and temperature was a better predictor of hornbill exhibit use than visitor presence. Behavior was significantly affected by the presence of keepers and individual variation in behavior was noted too. Visitor number mediated any interest in a keeper by birds: high visitor number decreased a bird's interest in its keeper. Whilst only a case study on a pair of birds, our research shows that any “visitor effect” is heavily influenced by other environmental variables and that different categories of human (i.e., visitor, keeper) affect how zoo animals utilize their environment.

## Introduction

For most zoo animals, the visitor presence is a normal part of their daily routine. Since the early 1970s, researchers have suggested that this visitor presence may play a role in modifying the behavior of the animals being observed (1, 2). As reviewed by Davey (3) the ‘visitor effect’ has been described in many early studies as a negative influence on animal behavior, decreasing maintenance behaviors or increasing aggressive interactions. Alternatively, research can also show no observable change in behavior with visitor presence or intensity (4, 5) and in some circumstances, the presence of visitors may be enriching (6), particularly where visitor- animal interaction has a positive outcome (2). For example, gentoo penguins (*Pygoscelis papua*) show increased pool usage and positive increases in behavioral diversity with increasing visitor number (7) and a long-billed corella (*Cacatua tenuirostris*) performed “attention-seeking” behaviors, such as bobbing up and down and dancing on the spot, directed at visitors standing at the bird's enclosure (4).

Considerable interest has also focused on the effect of keeper presence on the behavior of zoo animals (8). As daily providers of resources, the work of a zookeeper may be an enriching feature of the captive environment but, for some species, this human presence within their environment could be negative (9). For many mammals, the human-animal relationship that can develop between an animal and its keeper may be beneficial to the animal's welfare state (6). Experienced keepers are more likely to recognize potentially negative behavioral changes in mammalian charges compared to non-mammalian ones (10), and the increasing recognition of a keeper by the animal reduces the stress of having humans in and around the enclosure (11, 12).

Whilst there is considerable interest in assessing both visitor and keeper effects, studies may be limited. It is often difficult to determine a baseline behavior for a study subject, particularly in zoos that are open to the public every day (3). Evaluation of research findings may be further complicated by limited prior information on the species and researchers may find it tricky to determine which behaviors are indicative of changes in welfare state (13). Methods to measure the visitor effect include assessment of visitor number, noise and behavior (2)—different taxa will vary in their responses to these factors, creating difficulties when designing repeatable research projects (14).

Much of the existing visitor effects literature has a mammalian focus, with primates dominating (2, 15). Zoos house a multitude of non-primate, non-mammalian taxa that may also be affected by visitors and this diversity in zoo-housed taxa means that studies assessing the visitor effect across a wider-range species would be beneficial (3) to inform husbandry practices and welfare assessment.

As such, the aim of this research was to determine any influence of human presence on a representative species of commonly housed zoo bird, a hornbill (Bucerotidae). Species360's Zoological Information Management System (ZIMS) database identifies over 2,600 hornbills housed in global species360-registered institutions as of August 2019 (16). Hornbills remain challenging to breed in captivity (17), and further research would be beneficial to identify the impact of humans on captive hornbill behavior, and thus provide evidence that may help understand any further influences on reproduction. The species focus for this research was the black-casqued hornbill (*Ceratogymna atrata*), a large hornbill from Africa, known for its sophisticated cognitive capabilities (18) with a decreasing wild population trend (19). Wild black-casqued hornbills (hereon referred to as “hornbill”) are normally found in pairs (20), but small groups of up to five birds are frequent and congregations of up to 40 individuals have been found on fruiting trees (21). This species feeds on at least 19 species of fruit in the wild and also invertebrates as a supplementary food source (22). Given their flexible social system and diet, this hornbill is a relevant study subject for analyzing the relationship between variable husbandry influences (i.e., human presence), behavior, and welfare as have evolved to cope with a very heterogeneous, widely fluctuating environment. More widely, a general lack of research on captive hornbill behavior, coupled with their poor reproductive success in zoos (23–25) but need for conservation action due to declining wild populations (26, 27), makes study of hornbill behavior and welfare in the zoo of increasing relevance and importance.

## Materials and Methods

### Subjects and Study Design

Data collection took place from 13th to 31st August 19 at Blackpool Zoo, Lancashire, UK and 17 days of observation were conducted in total. A pair of hornbills (both ~1 year of age) were observed for 90 h. The hornbills were housed with a pair of blue cranes (*Anthropoides paradiseus*); the enclosure included an indoor area measuring ~3 m^3^ (containing feeding and drinking areas, heat lamp and perches) connected to an outdoor exhibit measuring ~20 m (length) × 8 m (width) and from 8 to 10 m in height. The outdoor section contained various furnishings, such as natural planting and perches (see a schematic illustration of the enclosure in Supplementary Figure 1). No interaction between the hornbills and the cranes was noted during the observation period, nor did the hornbills actively seek to avoid the presence of the cranes.

An ethogram (Table 1) was developed using previous research from Kozlowski et al. (17). Each day consisted of 6-h of observation: 10:00 to 12:00; 12:20 to 14:20; and 15:00 to 17:00. Each hour of each observation period was considered a separate sampling event for data analysis (see “Data analysis”). This timeframe enabled capture of the varying numbers of visitors across each day; from when visitors first started arriving at the Zoo through to when the vast majority leave. Due to the short period of observation, no data were collected during periods of time when visitors were not within the Zoo. State behaviors (*n* = 90 records per bird) were recorded using instantaneous focal sampling at 1-min intervals (28), as was the location of each bird (indoor or outdoor exhibit) within the enclosure. A total of 10,740 min of behavioral data were collected per bird.

**Table 1:** Ethogram of captive black-casqued hornbill state behaviors.

**Behavior**	**Description**
Preening	Using beak to manipulate feathers anywhere on the body. Normally carried out whilst perching and lasting longer than 10 s.
Allopreening	Using beak to manipulate the feathers of another individual bird, anywhere on its body.
Foraging	Picking up food items and/or water using the beak, head is tilted back or jolted upwards and slightly backwards, throwing items toward the back of the mouth. Includes swallowing of food items. Some vocalization may be made.
Standing	Terrestrial. No movement along ground, however minimal head and wing movement may occur. No direct interest toward anything specific. Some vocalization may be made.
Perching	Sat/stood on a branch or any structure off the ground. No movement along or around structure, however minimal head and wing movement may occur. Some vocalization may be made.
Sunbathing	Perched or stood, with wings spread open or drooping down slightly, may be leant out and showing back to heat source (likely to occur near or under a heat source or in sunny weather).
Locomotion	Ariel or terrestrial. Flying using wings. Or putting one foot in front of the other either along the ground or along a branch, to walk or hop.
Inactive	Perched or sat motionless with head resting on back of body, no interaction to other individuals or its surroundings.
Out of Sight	The animals are not visible to observer, and possibly most visitors outside the enclosure.

To explain potential behavioral changes associated with the number of visitors, the observer started recording the location of each bird from the third day onwards (*n* = 80 records/bird). Visitor number was counted each minute and the mean value was used to determine groupings into high, medium or low visitor number (high = mean of 13+ visitors/minute; medium = mean of 7–13/minute; low = mean of 1–7/minute) for each hour. Categorization was based how busy the viewing areas of the enclosure appeared to the observer and how much of the enclosure was visible to the observer when different numbers of people were gathered around. Visitors could look into the viewing window of the indoor enclosure but the viewing area was small; the inside house kept darker and more secluded for the hornbills, who could perch away from, and higher than, the main window so had the choice to be in or out of view (see Supplementary Figure 2). Hornbills had *ad lib* access to and from the indoor enclosure during the duration of the study. The presence or absence of keepers was also recorded each minute, for each observation period. Visitor and keeper presence were recorded in areas visible to the birds (within or around their enclosure), approximate distance of up to 20 meters away from enclosure out the front and visible side (near outdoor area of enclosure) and 10 meters out from back (other side of enclosure by the indoor area). The mean (±SD) number of visitors was 8.8 (±0.12), with the minimum being 1 person and the maximum being 51 people. For all periods of data collection, the observer was considered a visitor.

Local weather conditions (rain, cloudy, sunshine), temperature and humidity were recorded for the start of each observation hour using the Met Office website https://www.metoffice.gov.uk. For the overall study period, the mean (±SD) temperature was 17.01°C (±0.14) and humidity was 80.24% (± 0.90). The most common weather condition was cloudy.

### Data Analysis

Data were analyzed in R studio v. 1.2.19 (29). To analyze the potential effect of visitors, local weather conditions (including temperature and humidity), keeper presence, and individual bird ID on state behaviors and on time spent outside compared to inside, mixed effects models with date blocked as a random factor to account for the repeated measurements were run using the “lmertest” package in R (30). The “MuMIn” package (31) was used to calculate *r*^2^ values for each model run.

To determine any effect of temperature, humidity, and weather (cloudy, raining, or sunny) on daily visitor numbers at the hornbill enclosure, a general linear model was run. Anecdotally, zoo visitors are known to gather around an enclosure when they see a keeper working inside, and therefore Spearman's rho correlation was run on the time spent by a keeper in the enclosure and the mean number of visitors at the enclosure for that hour of observation. The same correlation was run to check any relationship between the temperature and humidity for each observation hour.

**Figure 1:**
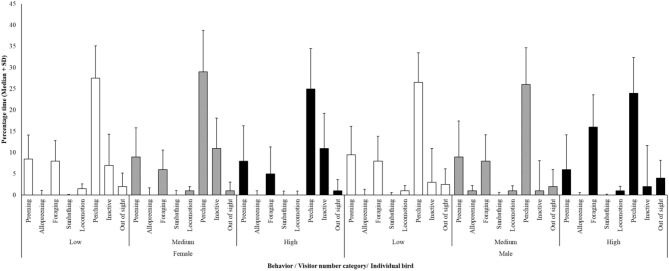
Average (± standard deviation) time-activity budget for the female and male hornbill against different categories of visitor number. White bars, low visitor number; Gray bars, medium visitor number; Black bars, high visitor number. Perching is the most commonly observed behavior and time spent perching is consistent between these two individuals and across visitor number categories.

Based on descriptive analysis (Figure 1), minutes spent preening (as a comfort behavior), out of sight (as a potential indicator of stress based on how the birds could hide themselves away in different areas around the inside and outside enclosure), inactive (as measure of limited behavioral diversity, i.e., birds spending the majority of their time inactive may not be performing a full daily time-activity budget) and foraging (as an exploratory behavior) per observation period per day were included as dependent variables. Out of sight (i.e., being away from visitors) and inactivity are noted in Nimon and Dalziel (4) as behavioral outcomes of different levels of visitor effect on another species of socially and cognitively complex bird, hence their inclusion here. Temperature was included in the modeling of preening, foraging or inactivity and visitor number, and for the time birds out of sight. The interaction between visitors and temperature was also included, as well-individual bird ID, and finally date (as the random factor).

To see whether time of day influenced these three behaviors and time out of sight, in conjunction with the visitor and temperature interaction, time of the observation was coded (morning from 10:00 to 12:00; noon from 12:00 to 13:50; afternoon from 13:50 to 17:00) and included in a further mixed effects model, again with date blocked as a random factor. Time codes were based on discussion between the three authors as to the most practical, biologically-relevant, and optimal for capturing change in visitor/keeper presence way of categorizing when observation occurred.

Output is presented from the anova (model name) function in RStudio. *Post-hoc* testing using the “lsmeans” and “pbkrtest” packages (32, 33) was run for behaviors were time code showed a significant relationship to change in activity. To unpick any impact of when keepers might be in with the birds during different times of the day, and hence changing bird activity, the mean number of minutes for each time category was calculated to see at what times of the day keepers were in with the hornbills for longest.

Interest from each bird (measured as the number of minutes that a bird looked in the direction of or moved toward the keeper or visitor) was included as the dependent variable in a mixed effects model to determine any influence of keeper presence (time in minutes at or in the enclosure), visitor number and individual bird ID, again with date blocked as a random factor.

Significant outputs from the generalized linear models run are provided in the results section with estimate ± standard deviation, *r*^2^ value, degrees of freedom, t value and *P*-value at the 5% level. For relevant non-significant results, *P*-values are given.

## Results

### State Behaviors

A time activity budget was developed to show the behavior of both the male and female hornbill under low, medium and high visitor numbers (Figure 1).

To investigate the relationship between visitors and weather, linear models were run to identify any significant predictors of increased visitors to the zoo. There is no effect of weather condition on visitor number (*P* = 0.731) but there is a significant influence of temperature and humidity. Significantly more visitors are present on hotter days (estimate = 1.25 ± 0.259; *df* = 175; *t*-value= 4.83; *P* < 0.001) and significantly fewer on more humid days (estimate = −0.198 ± 0.039; *df* = 175; *t*-value = −2.78; *P* = 0.006). Temperature increase showed a positive correlation with humidity (*n* = 26; *r* = 0.585; *P* = 0.002) and this was the justification for including temperature in the modeling of bird behavior / time out of sight and visitor number plus other relevant interactions (see “data analysis” section).

There are no significant effects on preening behavior for either hornbill (*P* = 0.956). There is an individual difference for time being out of sight (estimate = 1.044 ± 0.416; *r*^2^ = 37%; *df* = 159.6; *t*-value= 2.51; *P* = 0.013), which is significantly higher in the male bird than the female bird in this case. All other predictors of time spent out-of-sight are non-significant. The same difference is seen for foraging, with the male spending more time foraging than the female (estimate = 3.31 ± 0.814; *r*^2^ = 28%; *df* = 157.9; *t*-value= 4.09; *P* < 0.001). Again, there is no significant effect of visitors, temperature or the interaction between them on time spent foraging. Finally, the individual bird also significantly predicts, with the female hornbill spending more time inactive than the male (estimate = −4.82 ± 1.09; *r*^2^ = 28%; *df* = 159.6; *t*-value = −4.44; *P* < 0.001). All other potential predictors of inactivity were non-significant.

Time of day (category) does not significantly predict when hornbills would be preening [*F*_(2, 167.9)_ = 0.969; *r*^2^ = 19%; *P* = 0.381], foraging [*F*_(2, 166.9)_ = 0.163; *r*^2^ = 28%; *P* = 0.849], or out-of-sight [*F*_(2, 165.01)_ = 2.24; *r*^2^ = 38%; *P* = 0.109] but it does predict when these birds are likely to be inactive [*F*_(2, 168.3)_ = 3.2; *r*^2^ = 29%; *P* = 0.04]. Birds are more likely to be inactive in the afternoon compared to in the morning (estimate = 3.91 ± 1.58; *df* = 169; *t*-ratio = 2.48; *P* = 0.04), irrespective of temperature^*^visitor number (estimate = 0.101 ± 0.06; *df* = 172.8; *t*-value = 1.71; *P* = 0.09). Keepers were likely to visit the hornbill enclosure more frequently in the morning but stay for shorter times (*N* = 30; mean = 1.93 ± 0.14) compared to afternoon visits that were less frequent but longer in duration (*N* = 18; mean = 4.11 ± 1.13).

### Enclosure Occupancy

**Figure 2:**
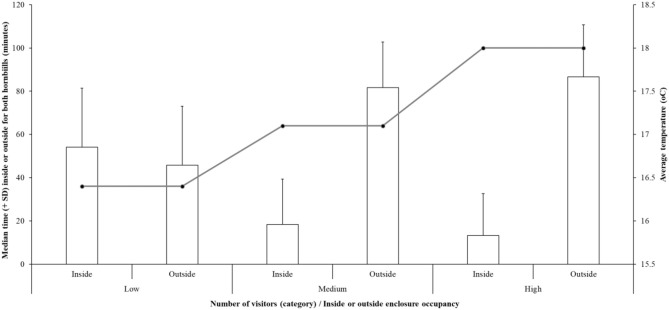
The time (for both birds combined) spent inside or outside (median number of minutes ± standard deviation) compared to the three categories of visitor number and average temperature for these visitor categories.

Modeling predictors of time spent outside for both hornbills including individual bird ID, visitor number, temperature, interest in visitors from the birds, and the relationship visitor number^*^ temperature shows an overall significant fit (estimate = 135.6 ± 30.23; *r*^2^ = 51%; *df* = 98.34; *t*-value = 4.473; *P* < 0.001; Figure 2). The effect of visitor number on time spent inside or outside is significant- an increasing number of visitors suggests less time inside for the birds (estimate = −7.18 ± 2.63; *df* = 151.84; *t*-value = −2.73; *P* = 0.007). However, as the relationship between visitor number and temperature is also significant, the cause of the hornbills being increasingly outside with higher visitor numbers is explained by higher temperatures (estimate = 0.313 ± 0.150; *df* = 151.24; *t*-value = 2.09; *P* = 0.039). There is no individual bird difference for time spent inside or outside (*P* = 0.189). Figure 2 illustrates the relationship between enclosure occupancy and temperature.

### Keeper and Visitor Presence and Hornbill Interest

**Figure 3:**
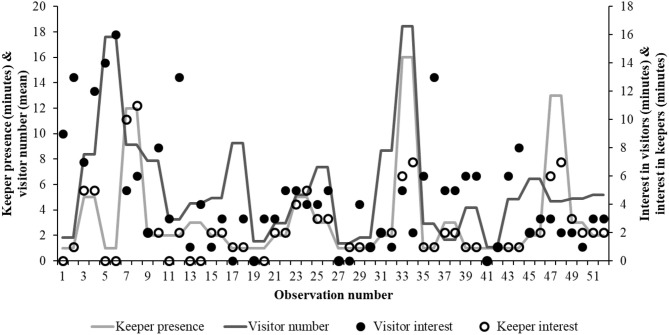
The relationship between the bird's interest in keepers and the visitor number (around the enclosure) and keeper time within and around the enclosure. As visitor number increases, the bird's interest in the keeper is shorter. The lack of relationship between visitor interest and visitor number suggest a role for the keeper in this human-animal relationship.

For 71% of all occurrences (*n* = 90) no keeper was present within or near the enclosure (Figure 3). Using these remaining data where a keeper was present, the birds' degree of interest in the keeper and in the visitors (based on minutes of observation from the hornbills) was analyzed. As keeper presence and visitor number correlated (*n* = 26; *r* = 0.442; *P* = 0.024) the interaction between keeper presence^*^visitor number on bird interest in both was included in the model.

There is a significant interaction between the presence of the keeper in or near the enclosure and an increasing interest from the bird (estimate = 0.864 ± 0.99; *r*^2^ = 85%; *df* = 9.43; *t*-value= 8.64; *P* < 0.001). There is no significant effect of bird interest in visitors (*P* = 0.419), visitor number (*P* = 0.395) and individual bird ID (*P* = 0.223). The interaction between visitor number^*^keeper presence is a significant factor (estimate = −0.026 ± 0.01; *df* = 27.44; *t*-value = −3.403; *P* = 0.19) on the hornbill's interest in the keeper, suggesting that birds become less interested in the keeper as visitor number grows (Figure 3).

When running this model for the bird's interest in visitors there is no effect of keeper presence (*P* = 0.937) and the interaction between visitor number^*^keeper presence also becomes non-significant (*P* = 0.546). Individual bird is significant (estimate = −1.76 ± 0.783; *r*^2^ = 50%; *df* = 36.71; *t*-value = −2.25; *P* = 0.03) with the female bird spending more time interested in visitors than the male (overall minutes of interest per observation period from the female 3.9 ± 0.34 and for the male 2.8 ± 0.26).

## Discussion

Overall, we identified no direct “visitor effect” on the behavior of this pair of hornbills and our results support recent findings, using a mammalian species (the ring-tailed lemur, *Lemur catta*) that the visitor effect may be overestimated (34) if other behavioral influences are not fully considered. For these hornbills, several other variables, such as the individual characteristics of the birds themselves, had greater influences on their behavior.

### Behavior

Significant differences in time-activity budgets were identified for these hornbills, with the male spending longer foraging and the female more time inactive. The female hornbill was less likely to be out-of-sight compared to the male. Time of day significantly predicted increases in activity, with the hornbills being more active in the morning compared to the afternoon. Time (minutes) that a keeper spent in the enclosure may be influencing activity, with the shorter, but more frequent morning visitors causing more interest from the birds in the daily husbandry routine. However, temporal changes in inactivity could be explained by the natural ecology of these birds- wild hornbills are known to use vocalization to organize social groups to move from roosting sites to foraging sites in the early morning (25). Expanding data collection into the earlier morning and later evening to capture husbandry influences on the birds (e.g., the provision of fresh food) would help unpick this complicated, multilayered relationship.

These hornbills rarely engaged in behaviors suggestive of pair bonding (e.g., allopreening), potentially because these birds are relatively young and recent arrivals to this Zoo. The female also showed significantly more interest in visitors and this may be related to the bird's inactivity and/or personality, as a bolder or less nervous individual may be less motivated to move away from visitors. Personality is known to affect how highly-cognitive species engage with human interactions in and around their enclosure (35, 36), so further assessment of bird personality traits alongside of state behavior data could be useful in explaining reactions to visitors and keepers. Well-established hornbill pairs spend more time involved in social behavior directed at their partner (17, 20). The immaturity of these study birds may have resulted in more interest in their surroundings compared to in each other.

There were no other significant impacts on hornbill time-activity budgets, aside from individual difference. Behaviors selected for further analysis—foraging and preening—are often used as welfare indicators for captive birds (37), and based on the results presented here, there are no marked changes in the performance of these behaviors under different conditions that we measured (i.e., low, moderate, and high visitor number). Perching, as the commonest behavior observed, was also consistent between conditions; measurement of where birds are perching (height and distance to or from visitors) could help further evaluate this behavior. Wild frugivorous hornbills predominantly forage in the upper and lower canopy (38) so perch height and food placement may influence overall time-activity patterns of captive birds. Large species of frugivorous hornbills are known to be selective in their foraging choices and can spend up to 60 min at a specific fruit source (39). Changes to how food is presented around the enclosure, e.g., multiple locations where birds need to work for a reward (25), may encourage more foraging and exploration time and reduce time spent perching or inactive in large hornbill species in captivity. Black-casqued hornbills are also known to wander extensively across a large feeding range (40), so increased enrichment and abilities for flight in their enclosures may reduce time spent inactive and perching.

### Enclosure Occupancy

Enclosure zone use is often linked to welfare assessment for zoo-housed animals, with avoidance of specific zones a potential inference of poorer welfare (41, 42). Our model including individual bird ID, climate and visitor numbers, explained 51% of the observed zone use for the hornbills. Initial analysis suggested that higher visitor numbers appeared to be associated with increased use of the outdoor exhibit. However, this relationship is less assured when climatic variables are included in the model. Consequently, consideration of all factors that influence animal behavior and enclosure usage is required when attempting to quantify any “visitor effect,” as other more fundamental reasons may produce a more robust, biologically-relevant explanation, of the individual's behavior.

This enclosure may have provided sufficient species-appropriate areas for these hornbills to have the choice to move away from visitors, therefore no avoidance behaviors (i.e., decreased enclosure zone occupancy or repeated, perch to perch hopping) were performed. Choice to be on or off show is known to correlate with the performance of behaviors that indicate improved welfare (43), so the lack of visitor effect may in part be due to the settled nature of the two hornbills in their exhibit and their ability to “cope” with visitor numbers. Further evaluation of enclosure usage alongside of time-activity patterns is required to fully understand the suitability of this aviary for these hornbills, however.

### The Visitor and Keeper Effect

Our results show that “visitor effect” is more complicated than it may appear, and the presence of visitors alone may be not the complete causative factor in changing the behaviors of these hornbills. Our results support Goodenough et al. (34), who show that weather and changes in time of day are stronger influences on zoo animal behavior than visitors themselves, and those of de Azevedo et al. (44) who show no visitor effect on behavior in another common zoo bird, the greater rhea (*Rhea americana*). Consequently, research into animal behavior, including visitors as an independent variable must factor in temporal and climatic changes. Further analysis of visitor behavior may help to identify the presence of possible visitor effects on animal welfare; for example via assessment of changes to the soundscape around the enclosure caused by visitors and any accompanying animal response (14, 45). Observation of the visitors by the author of this paper who conducted data collection (JS) noted that as visitor number increased, the immediate vicinity was generally noisier but overall, the hornbill's enclosure was in a “quieter part” of the Zoo. Personal observation also noted that visitors did not generally stop talking at the enclosure and in small to moderate groups, normal conservational noise levels were apparent. Visitors were also noted as walking past without stopping at the hornbills as if the enclosure had not been recognized.

There was a clear relationship between the minutes that a keeper was present around the birds and the birds' interest in the keeper. However, when visitor numbers were higher, the hornbills reduced their focus on keepers. This relationship is further complicated by the fact that there was no significant relationship between visitor number and visitor interest from birds. These birds maybe habituated to visitors (because visitors provide no benefit or threat), but that their presence remains a distraction when coupled with other stimuli. Distraction of bird behavior by human actions is noted in other literature in relation to noise levels and approach to nesting colonies in the wild from groups of tourists, e.g., negative impacts on hoatzin, *Opisthocomus hoazin*, activity (46) and other authors investigating “distraction” effects caused by human activities suggest that species can habituate over time (47), therefore such mechanisms may be at play in the captive environment too. If keepers spend less time in the enclosure when visitor numbers are higher, this may also impact on the bird's attention that is directed toward them. Measurement of the influence of visitor presence on keeper duties and time spent in an enclosure would be a useful follow up to this research. The positive correlation between visitor number and the keeper being present is also worthy of further investigation. Anecdotally, zoo visitors are drawn to an enclosure when a keeper is present as “something interesting might be happening” and therefore further adding to the dynamics of the interaction between the animal on display, its keeper(s) and how it perceives the influence of visitors.

As providers of resources, but also invaders of space, zookeepers may be a source of both enrichment and stress (6). Unlike visitors, keepers can enter an exhibit daily and therefore habituation by the animals may be difficult (8). Anticipatory behaviors, performed based on the timing of specific aspects of husbandry (e.g., feeding), can be indicative of underlying motivational states (48) and their performance may also impact on the keeper-animal relationship (i.e., more interest in the keeper if a positive outcome is expected). Husbandry variables influence the visitor effect on the behavior of mammalian species (49); there is considerable scope for determining such husbandry impacts on non-mammalian behavior under different visitor conditions to fully appreciate animal responses to the zoo environment.

### Future Directions

This is a case study on a pair of hornbills of one species at one zoological institution, therefore wide-scale application of these results is limited. We have only one measure of individual bird characteristic (sex) and further study onto the effects of animal personality on the potential of the visitor effect is recommended, this animal personality can predict differences in responses to zoo visitors in captive mammals (36). Multiple measurement of behavior across days when visitor number is low would provide stronger evidence for the link between environmental conditions and outside enclosure usage, and how visitor presence influences this. A proximal cause of behavior change in these birds may be visitor presence and without measure of temperature effects independent of visitor presence, the relationship remains complicated. Specialized species of birds (i.e., those evolved for particular environmental conditions) are noted as having especially aversive reactions to visitors when prevailing environmental conditions are not optimal (50). As is noted in mammalian research, fully pinpointing behavior change caused by visitor presence and then inferring welfare state from it remains a challenge (51). With taxa such as birds, where outward signs of personality and behavioral expression can be harder to judge than in mammals, the visitor-behavior-enclosure usage-welfare relationship could be even more challenging to unpick. Extending this research to other populations of this hornbill in other zoos would enable further analysis of husbandry and enclosure variables on behavior patterns and aviary usage. It is possible that indirect visitor effects may be more prevalent than current research suggests. In order to extend this question further, researchers should consider the following:

- Investigate animal behavior across a range of time periods and seasons to fully capture the influence of weather conditions on behavior and zone occupancy, and their relationship with visitor numbers.
- As zoos move away from single-species aviaries toward larger, mixed-species and/or walk-through exhibits (52) knowledge of any potential visitor effect on enclosure usage would be relevant to animal husbandry.
- Comparing remote (e.g., trail camera) and in-person data collection would enable evaluation of any observer effect on animal-to-visitor, animal-to-keeper, and animal-to-observer interaction.

## Conclusion

Our study identified no impact of visitors on hornbill behavior or enclosure use. Visitor number positively correlated with temperature and so temperature should be factored into future visitor studies to avoid overestimation of any visitor effect. Individual hornbill characteristic (e.g., sex) was a significant predictor of behaviors such as foraging and inactivity, whereas visitor and keeper presence and weather conditions were not. When more visitors were present at their exhibit, hornbills spent less time showing interest in their keepers, suggesting more complexity to the keeper-animal relationship in certain conditions. Further research into both visitor and observer effects, across a range of hornbill species, over different seasons, and in different exhibit styles is recommended.

## Data Availability Statement

The datasets generated for this study are available on request to the corresponding author.

## Ethics Statement

This animal study was reviewed and approved by Sparsholt College Hampshire Ethics Committee.

## Author Contributions

JS: data collection and methods. PR: writing and data analysis. JB: editing and fact checking plus supervision of data collection.

## Conflict of Interest

The authors declare that the research was conducted in the absence of any commercial or financial relationships that could be construed as a potential conflict of interest.

## Supplementary Material

Supplementary Figure 1 Schematic plan (not to scale) of the hornbill enclosure at Blackpool Zoo.

Supplementary Figure 2 Hornbill indoor housing with visitor viewing area (photo credit: J. Scales).
