# Supplementary figures and images for: Why the “Visitor Effect” Is Complicated. Unraveling Individual Animal, Visitor Number, and Climatic Influences on Behavior, Space Use and Interactions With Keepers—A Case Study on Captive Hornbills
Source: Front Vet Sci. 2020 Apr 28;7:236. doi: 10.3389/fvets.2020.00236 (PMC7199352; doi:10.3389/fvets.2020.00236)

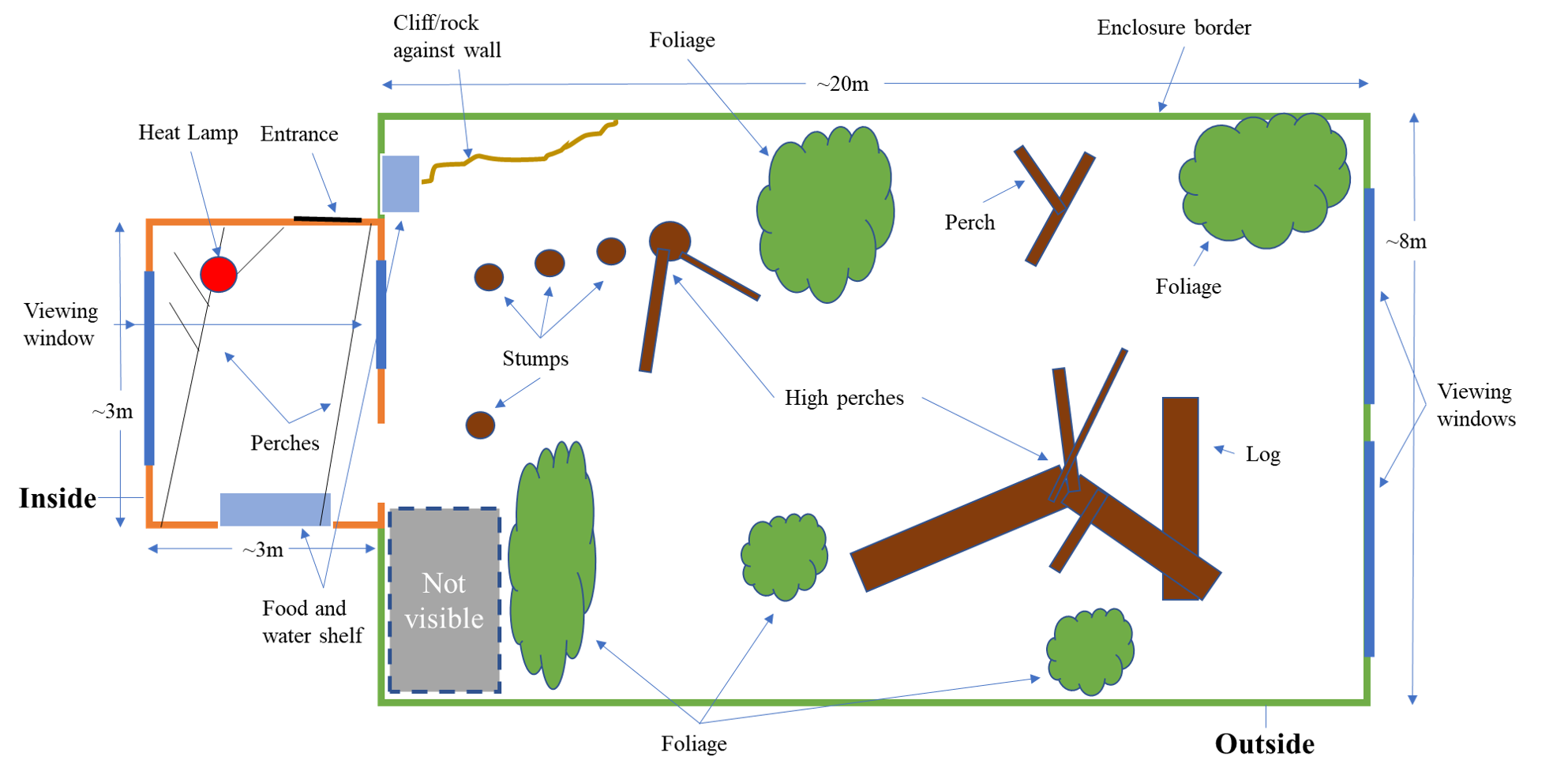

Supplement: Supplementary Figure 1 — Schematic plan (not to scale) of the hornbill enclosure at Blackpool Zoo. [file Image_1.tif]

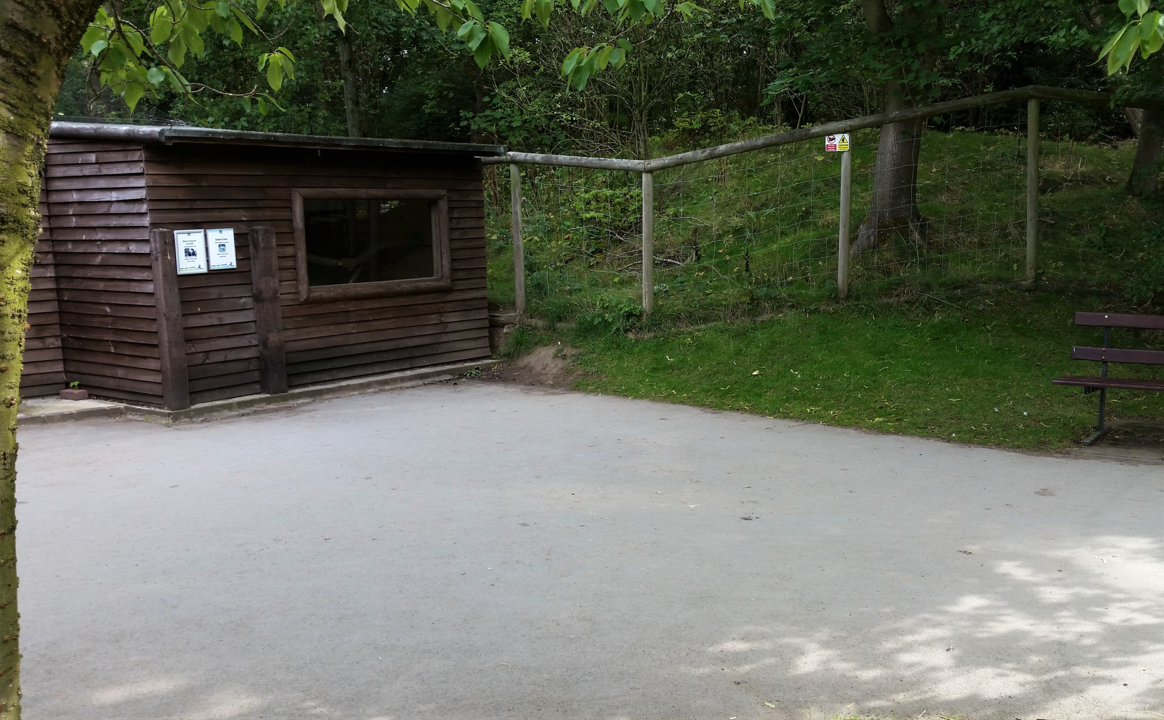

Supplement: Supplementary Figure 2 — Hornbill indoor housing with visitor viewing area (photo credit: J. Scales). [file Image_2.tif]
